# Identification of tumor-specific T cell signature predicting cancer immunotherapy response in bladder cancer by multi-omics analysis and experimental verification

**DOI:** 10.1186/s12935-024-03447-6

**Published:** 2024-07-20

**Authors:** Xiufeng Liu, Chujun Chen, Jiashan Li, Linna Li, Meng Ma

**Affiliations:** 1 State Key Laboratory of Oncology in South China, Collaborative Innovation Center for Cancer Medicine, Sun Yat-sen University Cancer Center, Guangzhou, 510080 People’s Republic of China; 2 Key Specialty of Clinical Pharmacy, The First Affiliated Hospital of Guangdong Pharmaceutical University, Guangzhou, 510080 People’s Republic of China; 3 Department of ultrasound medicine, Jieshou People’s Hospital, 339 Renmin Road, Jieshou, Fuyang, Anhui 236500 China

**Keywords:** Bladder cancer, Tumor-specific T cells, Prognostic signature, Immunotherapy, Multi-omics analysis

## Abstract

**Background:**

Numerous gene signatures predicting the prognosis of bladder cancer have been identified. However, a tumor-specific T cell signature related to immunotherapy response in bladder cancer remains under investigation.

**Methods:**

Single-cell RNA and TCR sequencing from the Gene expression omnibus (GEO) database were used to identify tumor-specific T cell-related genes in bladder cancer. Subsequently, we constructed a tumor-specific T cell signature (TstcSig) and validated its clinical relevance for predicting immunotherapy response in multiple immunotherapy cohorts. Further analyses explored the immune characteristics of TstcSig in bladder cancer patients from other cohorts in the TCGA and GEO databases. Western blot (WB), multicolor immunofluorescence (MIF), qRT-PCR and flow cytometry assays were performed to validate the results of bioinformatics analysis.

**Results:**

The established TstcSig, based on five tumor-specific T cell-related genes, could predict outcomes in a bladder cancer immunotherapy cohort. This was verified using two additional immunotherapy cohorts and showed better predictive performance compared to 109 published T cell signatures. TstcSig was strongly correlated with immune characteristics such as immune checkpoint gene expression, tumor mutation burden, and T cell infiltration, as validated by single-cell and spatial transcriptomics datasets. Notably, the positive correlation between TstcSig and T cell infiltration was confirmed in the TCGA cohort. Furthermore, pan-cancer analysis demonstrated the heterogeneity of the prognostic value of TstcSig. Tumor-specific T cells highly expressed *CD27*, *IFNG, GZMB* and *CXCL13* and secreted more effector cytokines for tumor cell killing, as validated experimentally.

**Conclusion:**

We developed a five-gene signature (including *VAMP5, TIGIT, LCK, CD27* and *CACYBP*) based on tumor-specific T cell-related genes to predict the immunotherapy response in bladder cancer patients.

**Supplementary Information:**

The online version contains supplementary material available at 10.1186/s12935-024-03447-6.

## Introduction

Bladder cancer is one of the ten most prevalent malignancies worldwide, with the majority of these being non-muscle invasive bladder cancer and the remaining 25% being muscle invasive bladder cancer [1].Currently, bladder cancer is primarily treated surgically, supplemented by radiotherapy and chemotherapy [2]. Advanced bladder cancer patients have a high mortality rate due to tumor recurrence or metastasis. Recently, the clinical efficacy and safety of immunotherapy for advanced bladder cancer have been demonstrated in several clinical studies [3–7]. However, immunotherapy strategies are not suitable for all bladder cancer patients, and many do not benefit from this therapy. Therefore, the critical question is how to precisely select bladder cancer patients who might benefit from immunotherapy.

T cell signatures have been developed in many cancers for predicting prognosis and immunotherapy responds using bioinformatics analysis. D Q Cai et al. developed a chemoresistant TILCD8T signature (cr-TILCD8TSig) to predict prognosis, chemotherapy response, and immunotherapy outcomes in patients with breast cancer [8]. Xiaomeng Zhao et al. constructed a regulatory T cell-associated gene signature to predict colon adenocarcinoma prognosis [9]. Karlijn Hummelink et al. identified a dysfunctional T-cell gene signature for predicting nonresponse to PD1 blockade in non-small cell lung cancer [10]. It is well-known that tumor-specific T cells play a critical role in effective anti-tumor immunity. Recent studies have demonstrated that tumor-specific T cells are associated with improved response and prognosis after immunotherapy, especially those activated by immune checkpoint inhibitors [11–13]. The presence of existing tumor-specific T cells is clearly necessary for efficacy of immunotherapy [14, 15]. Despite the heterogeneity of tumors, more and more signatures and biomarkers of tumor-specific T cells and neoantigen-reactive T cells have been identified in cancers [16–19], while a tumor-specific T cell signature related of bladder cancer is still unclear. Given the roles of tumor-specific T cells in cancer immunotherapy, constructing a tumor-specific T cell signature may help to provide biomarkers for predicting immunotherapy response and prognosis and elucidate underlying mechanisms involved in anti-tumor immune response.

Collectively, the purpose of this study is to identify and establish a tumor-specific T cell signature (TstcSig) to predict the outcomes of immunotherapy in bladder cancer. Hopefully, our work will help elucidate the transcriptional characteristics of tumor-specific T cells and precisely select bladder cancer patients who might benefit from immunotherapy.

## Method and material

### Datasets

Single-cell RNA and paired T cell receptor (TCR) sequencing data (GSE149652) [20] of T cells from human bladder tumors and non-malignant tissues were downloaded from the Gene expression omnibus (GEO) database and used to screen for tumor-specific T cell-related genes in bladder cancer. The gene expression and clinical data from the IMvigor210 cohort [21] were obtained from the IMvigor210CoreBiologies package and used to construct the tumor-specific T cell signature (TstcSig). Another independent bladder cancer immunotherapy cohort (GSE176307) [20] was downloaded from the GEO database to validate the prognostic capability of TstcSig. Additionally, raw data from an immunotherapy cohort of melanoma from the PRJEB23709 cohort [22] were downloaded from the EMBL-EBI database (https://www.ebi.ac.uk/arrayexpress/) and quantified using Salmon software [23] with the reference genome GRCh38. To further validate the correlation between TstcSig and T cell infiltration, single-cell RNA sequencing (GSE145281) [24] and spatial transcriptome data (GSE171351) [25] for bladder cancer tissues were obtained from the GEO database. The bulk tumor transcriptome and clinical data for 33 cancer types were downloaded from The Cancer Genome Atlas (TCGA) database.

### Data processing

We performed single-cell RNA sequencing data analysis by using Seurat R package [26]. The Harmony package (https://github.com/immunogenomics/harmony) [27] was used for data integration and batch correction. Initially, low-quality cells were excluded. The data were then normalized by using the ‘NormalizeData’ function, and the top 2,000 most variable genes were identified using the ‘FindVariableFeatures’ function. The data matrix was scaled and centered by using the ‘ScaleData’ function, and principal component analysis (PCA) was performed on the top 2,000 variably expressed genes to identify the top 50 principal components using the ‘RunPCA’ function. The Harmony package was then employed to integrate all datasets to eliminate batch effects. The ‘RunUMAP’, ‘FindNeighbors’, and ‘FindClusters’ functions of Seurat were applied for dimensional reduction and clustering analyses. Uniform Manifold Approximation and Projection (UMAP) was used for data visualization. TCRs that were obtained in tumors but not in normal tissues were considered tumor-specific TCR. Tumor-infiltrating T cells with tumor-specific TCRs were defined as tumor-specific T cells. For spatial transcriptomics analysis, processed data were obtained from the GEO database. Downstream data were visualized using ggplot2 R package (https://github.com/tidyverse/ggplot2).

### Differentially expressed genes and pathway enrichment analysis

For bulk RNA sequencing data, differentially expressed genes (DEGs) were identified using the limma R package [28]. Genes with |log fold change| > 1 and adjusted *p* value < 0.05 were defined as DEGs. For single-cell RNA sequencing data, DEGs were identified by using the ‘FindMarkers’ function of the Seurat R package, and genes with |log fold change| > 0.25 and adjusted *p* value < 0.05 were selected as DEGs.

Gene Ontology (GO) enrichment analysis and Kyoto Encyclopedia of Genes and Genomes (KEGG) pathway analysis were performed using clusterProfiler R package [29]. Pathways with adjusted *p* value < 0.05 was considered significantly enriched.

### Immune cell infiltration and ESTIMATE analysis

Tumor infiltrating immune cell proportions were calculated using IOBR R package [30] based on the TIMER, xCell, quanTIseq, and MCP-counter methods. The relative abundance of each infiltrating immune cell in the tumor microenvironment (TME) was visualized with heatmap diagrams. We calculated the ESTIMATE, immune, and stromal scores, and tumor purity using the ESTIMATE method within the IOBR R package.

### Gene sets variation analysis (GSVA)

Using the GSVA R package [31], we utilized the gene set variation analysis (GSVA) method to calculate the GSVA score of the tumor-specific T cell gene set. For single-cell RNA sequencing, TstcSig scores were computed using the tumor-specific T cells gene set as input to the AddModuleScore function from the Seurat R package. The scores of 109 published gene signatures [18] were calculated using the GSVA R package in three immunotherapy cohorts.

### Cell co-culture

Human peripheral blood mononuclear cells (PBMCs) were isolated from the blood of healthy donors. In the co-culture setup, T24 cell lines derived from human ladder cancer were placed in the upper chamber, while PBMCs were placed in the lower chamber. They were co-cultured in RPMI-1640 medium enriched with 10% fetal bovine serum and 300 IU/ml IL-2 for 3 days.

### Quantitative real-time PCR

Total RNA from tumor-specific T cells was extracted using the Tissue RNA Purification Kit Plus (ESscience) according to the manufacturer’s protocol. RNA qualification and quality control were performed in triplicate using a NanoDrop2000 spectrophotometer (Thermo Scientific). In 20 µl PCR mix, 1 mg of total RNA was reverse transcribed into cDNA using the RT001 Fast Reverse Transcription kit (ESscience) for qPCR. Quantitative real-time PCR was performed using a ArtiCanATM SYBR qPCR Mix (TSINGKE). The mRNA expression levels of CD27, CXCL13, ENTPD1, IFNG, GZMB and GADPH were determined using specific primers (Table 1).

**Table 1:** The primer sequences used in this study

Gene symbol	Forward primer	Reverse primer
CD27	CAGAGAGGCACTACTGGGCT	CGGTATGCAAGGATCACACTG
IFNG	TCGGTAACTGACTTGAATGTCCA	TCGCTTCCCTGTTTTAGCTGC
GZMB	CCCTGGGAAAACACTCACACA	GCACAACTCAATGGTACTGTCG
CXCL13	GCTTGAGGTGTAGATGTGTCC	CCCACGGGGCAAGATTTGAA

### Western blot analysis

Cell proteins were isolated using Radioimmunoprecipitation assay (RIPA) buffer. Western blot analysis was conducted following the manufacturer’s instructions. Polyvinylidene fluoride (PVDF) membranes were incubated overnight at 4 ◦C with CD27 and GAPDH antibodies, followed by incubation with horseradish peroxidase-coupled secondary antibodies. The western blot bands were analyzed using Image J software.

### Flow cytometry

Single cells were stained with monoclonal antibodies (mAbs) directed against CD3, CD4, CD8, IFN-γ, Granzyme B, and PDCD1, according to the manufacturer’s instructions. Dead cells were excluded using Fixable Viability Stain. Intracellular cytokine staining for IFN-γ and Granzyme B was performed using the Fixation/Permeabilization Solution Kit (BD Biosciences). Flow cytometry was conducted using Beckman (CytoFlex, USA), and data were analyzed using CytExpert software (CytExpert, Beckman, USA).

### Multicolor immunofluorescence

Cell suspensions were applied onto glass slides treated with polylysine (Sigma-Aldrich). Multicolor immunofluorescence was performed using antibodies against CD8. Image processing was conducted using Phenochart software (Akoya).

### Statistical analysis

All statistical calculations and visualization were performed using R software. Univariate Cox regression analysis was conducted using the survival R package (https://github.com/therneau/survival) to investigate correlations between TstcSig scores and the overall survival (OS) and progression-free survival (PFS) of patients with bladder cancer in three immunotherapy cohorts and the TCGA dataset. Bladder cancer patients were classified into high-TstcSig and low-TstcSig score groups based on the median TstcSig score. The Kaplan-Meier method was used to generate survival curves, and the log-rank test was used to determine statistically significant differences using the survminer R package (https://github.com/kassambara/survminer). The correlation between continuous variables was evaluated by Pearson correlation analysis. Unpaired Wilcoxon tests or Student’s t-tests were used for comparisons between two groups, and the Kruskal-Wallis test was used for comparisons among more than two groups. A *p value* < 0.05 was considered significant in all statistical methods. Statistical significance was recorded as follows: * *p* < 0.05; ** *p* < 0.01; *** *p* < 0.001; **** *p* < 0.0001; ns, nonsignificant.

## Results

### Identification of tumor-specific T cells related genes

**Fig. 1:**
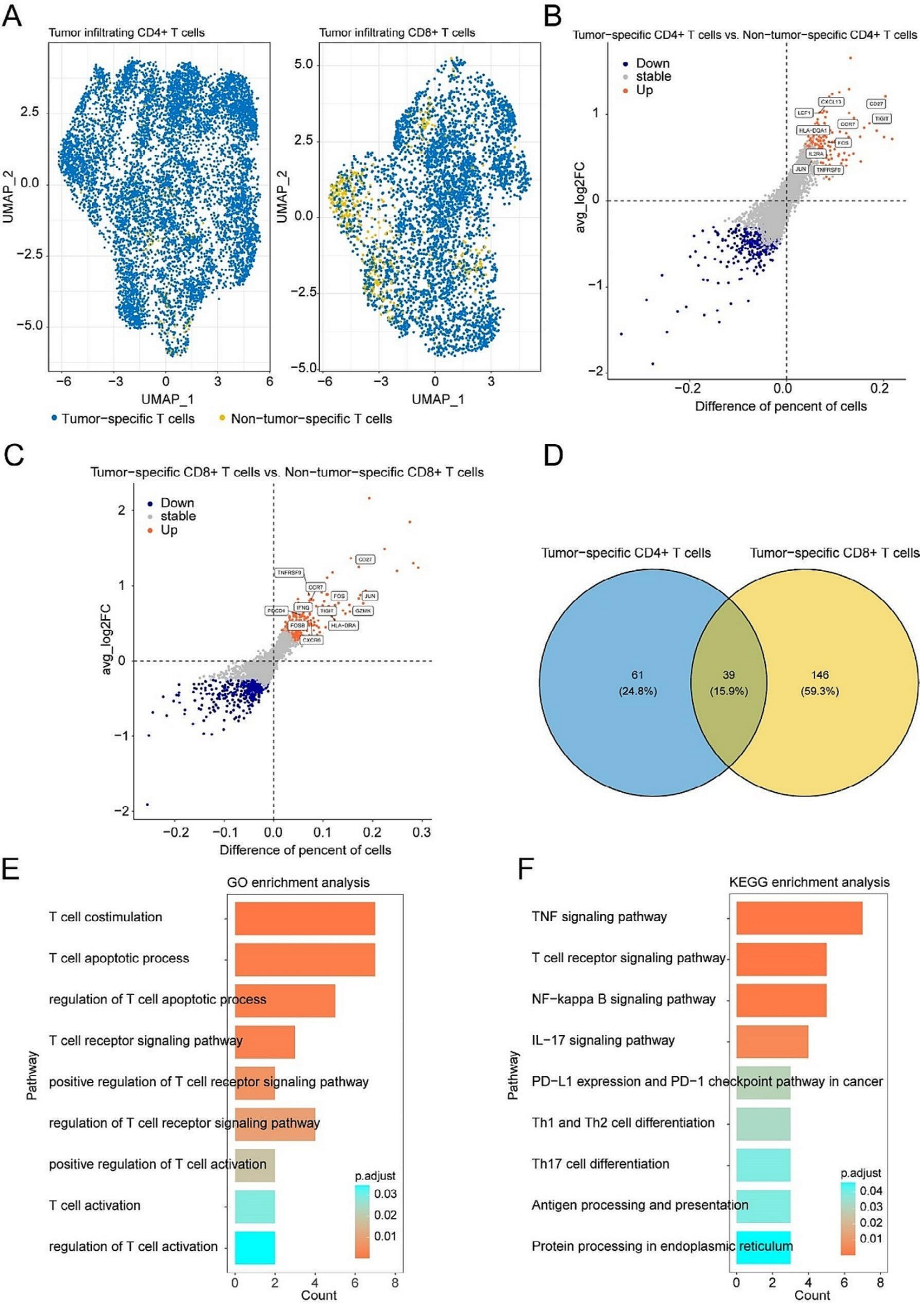
Identification of tumor specific T cell-related genes using single-cell RNA-sequencing analysis. (**A**) UMAP plots colored by different T cell types in infiltrating CD4 + T cells (left) and infiltrating CD8 + T cells (right). Blue: tumor specific T cells; Yellow: non-tumor specific T cells. (**B-C**) Dot plot showing the different expression genes in infiltrating CD4 + T cells (**B**) and infiltrating CD8 + T cells (**C**) (tumor specific T cells vs. non-tumor specific T cells). Blue: down-regulated genes; Grey: stable genes; Red: up-regulated genes. Genes of interest are labeled. (**D**) Venn diagrams showing the up-regulated genes of tumor specific T cells in infiltrating CD4 + T cells and infiltrating CD8 + T cells. The number in the overlapping area represents common up-regulated genes between the groups. (**E-F**) Bar plots showing the Gene Ontology (**E**) and KEGG (**F**) enrichment analysis results of 39 up-regulated genes

Given the crucial role of T cells in anti-tumor immunity, we first explored the transcriptional characteristics of T cells in tumors and its adjacent tissues using single-cell RNA sequencing (scRNA-seq) and TCR sequencing (TCR-seq), identifying tumor- specific T cells in bladder cancer (Fig. 1A). We then compared the expression profiles of tumor-specific CD4 + T cells and non-tumor-specific CD4 + T cells, as well as tumor-specific CD8 + T cells and non-tumor-specific CD8 + T cells. Tumor-specific CD4 + T cells showed higher expression levels of chemokine CXCL13, immune regulators FOS and JUN, and exhaustion markers TIGIT (Fig. 1B). Tumor-specific CD8 + T cells exhibited higher expression levels of cytotoxicity-related genes IFNG and GZMK, immune regulators FOS and JUN, and exhaustion markers TIGIT and PDCD1 (Fig. 1C). As shown in Figs. 1D and 39 hub genes were up-regulated in both tumor-specific CD4 + T cells and tumor-specific CD8 + T cells. GO analysis indicated that these 39 hub genes were primarily related to T cell activation pathways (Fig. 1E). KEGG analysis also revealed that the PD-L1 expression and PD1 checkpoint pathway in cancer were enriched with these 39 hub genes (Fig. 1F).

**Fig. 2:**
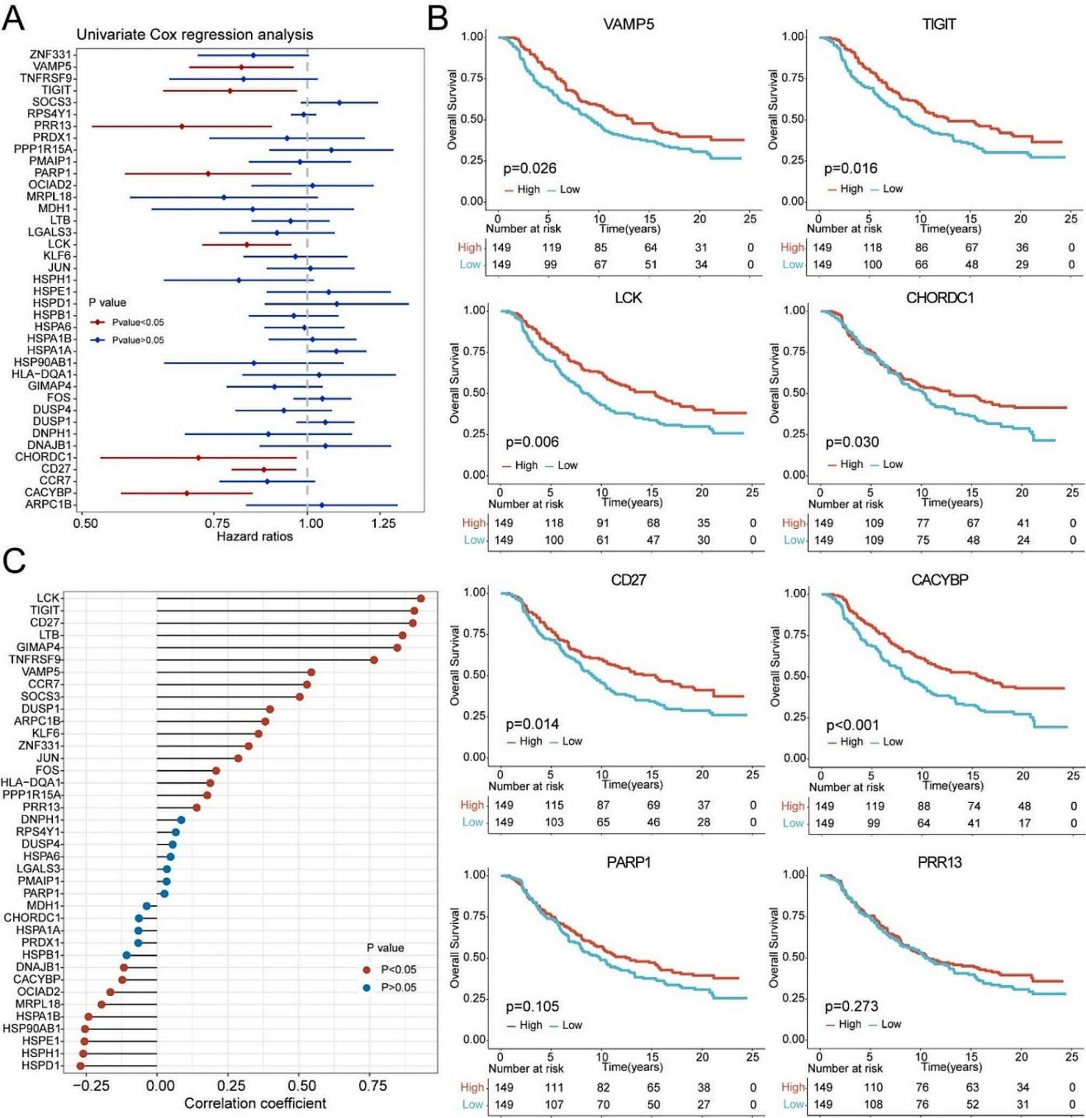
Identification of prognostic-related hub genes and correlation between T cell infiltration and genes expression. (**A**) Forest plot displaying the results of a univariate analysis. Variables with a Cox P value less than 0.05 are VAMP5, TIGIT, LCK, CHORDC1, CD27, CACYBP, PARP1, and PRR13. (**B**) Kaplan-Meier curves of survival analysis in bladder cancer patients treated with immunotherapy. Variables with a K-M analysis P value less than 0.05 are VAMP5, TIGIT, LCK, CHORDC1, CD27, and CACYBP. (**C**) Correlation analysis on T cell infiltration and 39 hub genes in the IMvigor210 cohorts

To further identified prognostic genes, we performed a univariate Cox regression analysis in the IMvigor210 cohort and found that 8 tumor-specific T cell-related genes were significantly associated with overall survival (OS) in bladder cancer patients (Fig. 2A). Furthermore, Kaplan-Meier survival analysis revealed that the higher expression of VAMP5, TIGIT, LCK, CHORDC1, CD27, and CACYBP was correlated with better outcomes in bladder cancer patients treated with immunotherapy (Fig. 2B). We further explored the association between 39 hub genes and T cell infiltration, finding that VAMP5, TIGIT, LCK, CD27 and CACYBP were significantly correlated with T cell infiltration (Fig. 2C).

### Tumor-specific T cell signature related to anti-tumor immunity and immunotherapy response

**Fig. 3:**
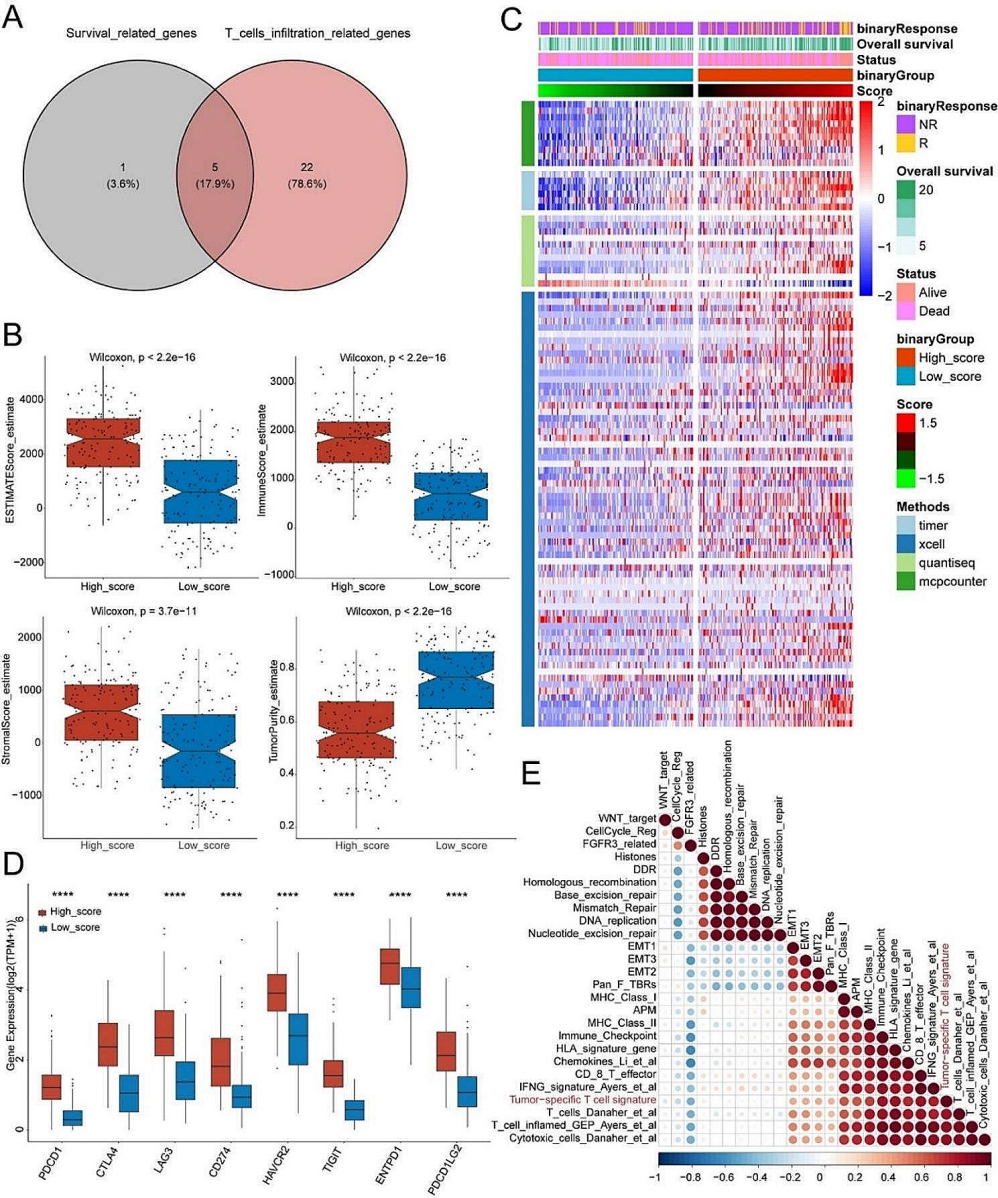
Tumor specific T cell signature associated with anti-tumor immunity. (**A**) Venn diagrams showing 5 hub genes correlating with prognosis and T cell infiltration in patients treated with immunotherapy. (**B**) Differences in ESTIMATE, immune and stromal score, and tumor purity between high- and low-score groups. (**C**) Heatmap showing the abundance of each TME infiltrating cell in patients treated with immunotherapy from the IMvigor210 cohort using TIMER, xCell, quanTIseq and MCPcounter methods. (**D**) Comparison of immune checkpoint expression between high- and low- score groups. **** represents the *p* values < 0.0001. (**E**) Heatmap showing the correlation between tumor-specific T cell signature and known gene signatures in the IMvigor210 cohort. Blue represents negative correlation and red represents positive correlation; larger the circle, higher the correlation

Then, we constructed a TstcSig based on five tumor-specific T cell-related genes associated with survival and T cell infiltration (Fig. 3A). The ESTIMATE, immune, and stromal scores of the high TstcSig group were significantly higher than those of the low TstcSig group, and the tumor purity of the high TstcSig group was significantly lower (Fig. 3B). Analysis of infiltrating immune cells revealed that high TstcSig patients had more immune cell infiltration, while low TstcSig patients had less (Fig. 3C). Additionally, immune checkpoint genes such as PDCD1, CTLA4, and CD274 were significantly higher expressed in the high TstcSig group compared to the low TstcSig group (Fig. 3D), indicating that TstcSig is associated with immunotherapy effectiveness. Further analysis showed that TstcSig was positively correlated with immune checkpoints, MHC class I, MHC class II, EMT1, EMT2, EMT3, IFNG signature, CD8 T effector, APM, chemokines, cytotoxic cells, and T cells inflamed scores, and negatively correlated with FGFR3-related scores (Fig. 3E). These results revealed that TstcSig was positively correlated with immune cell infiltration levels and played an important role in anti-tumor immunity.

**Fig. 4:**
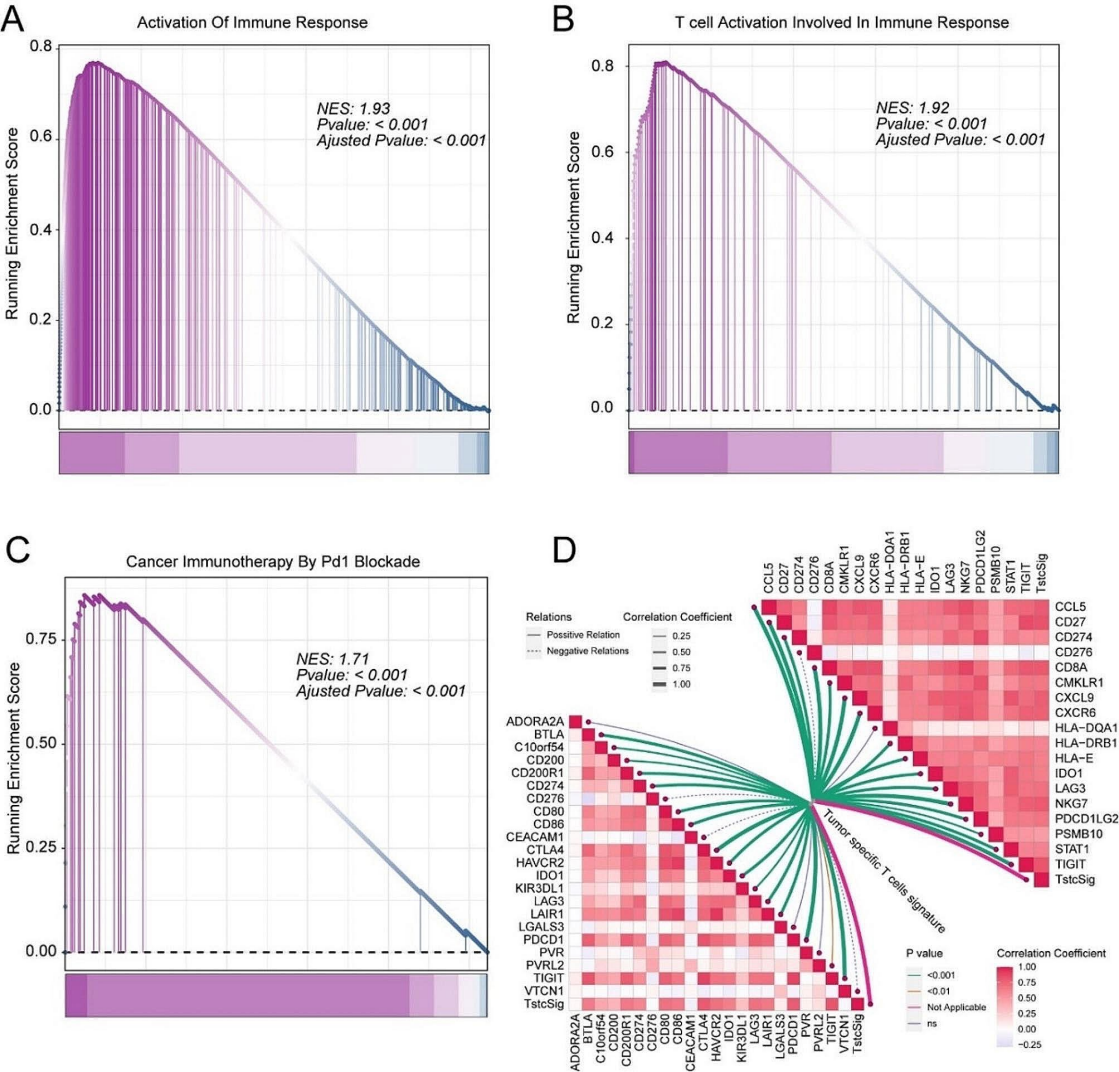
Results of functional enrichment analysis and the correlation between tumor-specific T cell signature and immunotherapy-related genes. (**A**) GSEA results of activation of immune response pathways in the TstcSig-high vs. TstcSig-low group in the MIvigor210 cohort. (**B**) GSEA results of T cell acitivation involved in immune response pathways in the TstcSig-high vs. TstcSig-low group in the MIvigor210 cohort. (**C**) GSEA results of cancer immunotherapy by PD1 blockade in the TstcSig-high vs. TstcSig-low group in the MIvigor210 cohort. (**D**) Correlation analysis of tumor specific T cells signature and inhibitory immune checkpoints (left) and T cell-inflamed score (right)

Gene set enrichment analysis (GSEA) was performed to identify detailed enrichment pathways based on the fold change values of all genes in the IMvigor210 cohort between the high TstcSig group and low TstcSig group. Immune response-related pathways including activation of immune response, T cell activation involved in immune response, and cancer immunotherapy by PD1 blockade, were up-regulated in the high TstcSig group (Fig. 4A-C). Notably, the TstcSig score correlated with T cell inflamed-related genes and immunotherapy-associated genes, showing a positive relationship with inhibitory immune checkpoints and T cell inflamed-related genes (Fig. 4D), suggesting that TstcSig is associated with the efficacy of immunotherapy.

### TstcSig predicts immunotherapy response and its potential biological role

**Fig. 5:**
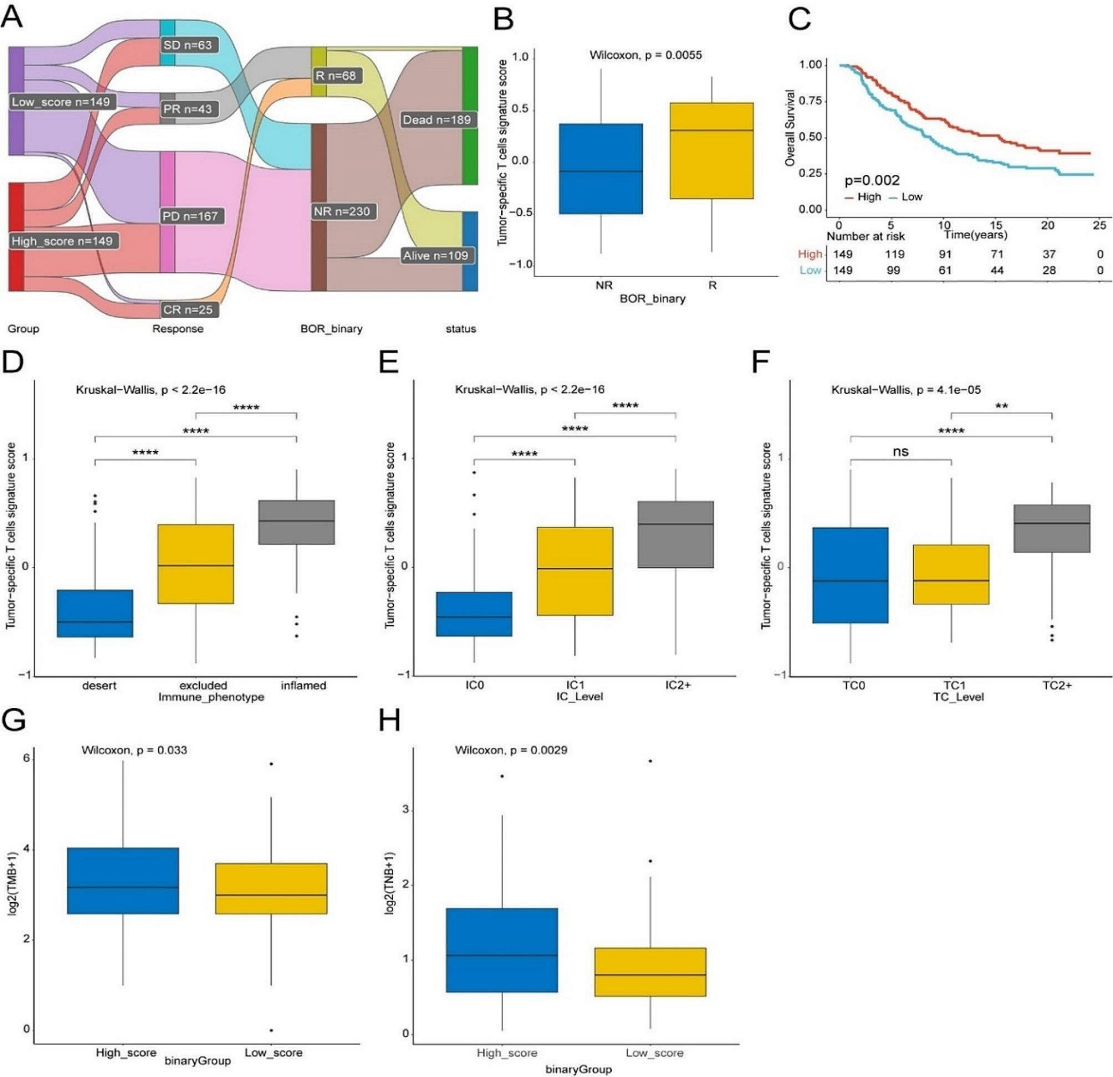
Potential biological role of the TstcSig as a predictor. (**A**) Sankey diagram showing the immunotherapy response and status in TstcSig high- and low-score patients from IMvigor210 cohort. (**B**) Box plot showing the relationship between TstcSig score and immunotherapy response. R: Responder; NR: Non-responder. (**C**) Kaplan-Meier curve showing the effect of TstcSig on overall survival in IMvigor210 cohort. (**D-H**) Box plots showing TstcSig score and immunophenotype (**D**), PD-L1 expression on tumor infiltrating immune cells (IC) (**E**), tumor cells (TC) (**F**), TMB (**G**), and TNB (**H**). NS, nonsignificant; ** represents *p* < 0.01; and **** represents *p* < 0.0001

We investigated the association between TstcSig and the efficacy of immunotherapy in the IMvigor210 cohort (Fig. 5A). The TstcSig score was significantly higher in the respond group compared to the non-response group (Fig. 5B). A positive association was found between survival and the TstcSig score in the IMvigor210 cohort (Fig. 5C). Our data further indicated that TstcSig was significantly associated with immunophenotypes and PD-L1 expression on immune cells (IC level) and tumor cells (TC level) (Fig. 5D-F). Given that tumor-mutation burden (TMB) and tumor neoantigen burden (TNB) are positively associated with the benefit of immunotherapy [32, 33], we compared the TMB and TNB levels between the high and low TstcSig groups. Results revealed that TMB and TNB were significantly higher in the high TstcSig group compared to low TstcSig group (Fig. 5G-H). These findings suggested that TstcSig not only can reflect tumor immunogenicity and predict the immunotherapy response in bladder cancer.

**Fig. 6:**
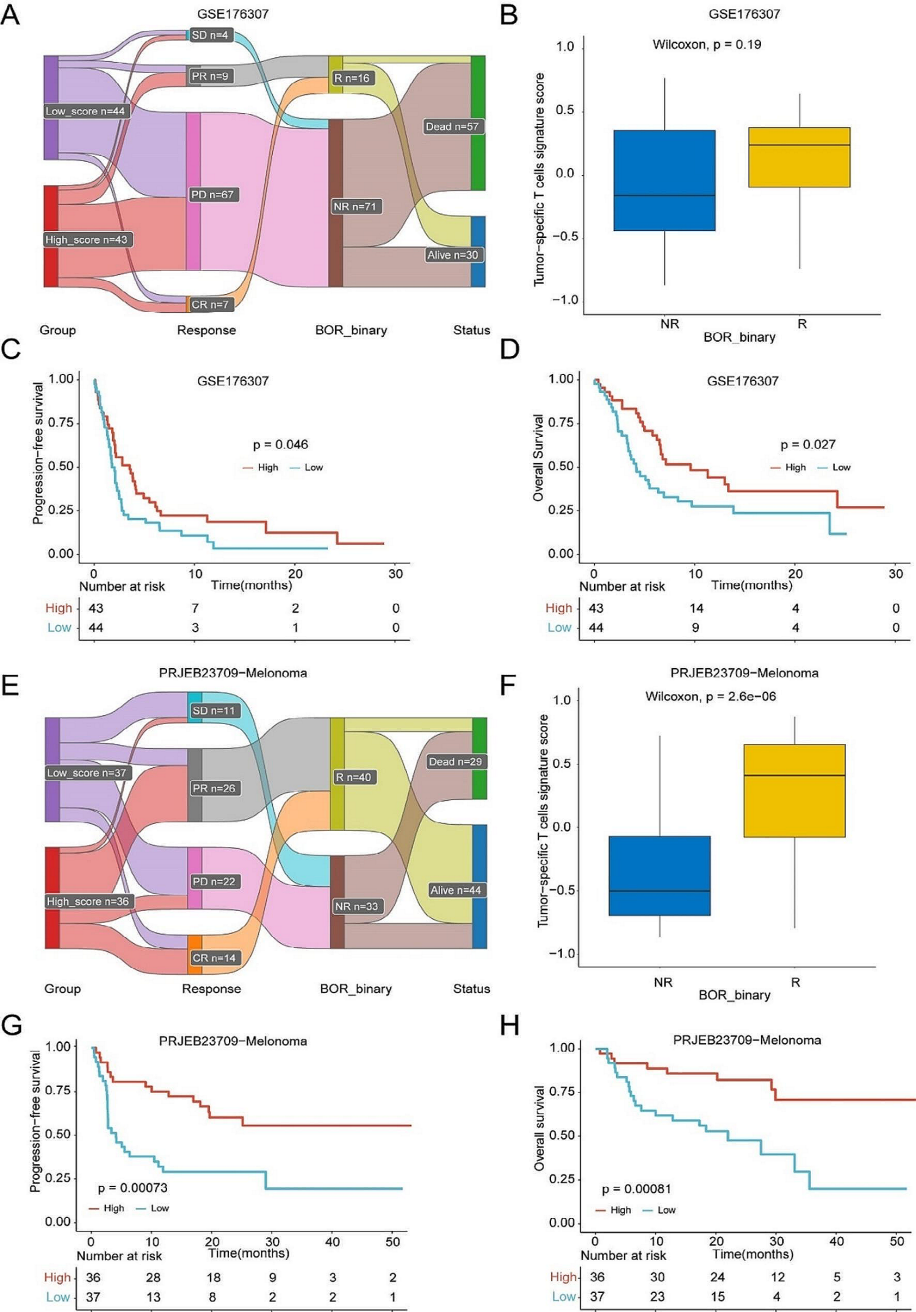
Verification of the immunotherapy prognostic value of TstcSig in multiple immunotherapy cohorts. (**A**) Sankey diagram showing the immunotherapy response and status in TstcSig high- and low-score patients from the GSE176307 cohort. (**B**) Box plot showing the relationship between TstcSig score and immunotherapy response in the GSE176307 cohort. (**C-D**) Kaplan-Meier curve showing the effect of TstcSig on progression-free survival (**C**) and overall survival (**D**) in the GSE176307 cohort. (**E**) Sankey diagram showing the immunotherapy response and status in TstcSig high- and low-score patients from the PRJEB23709 cohort. (**F**) Box plot showing the relationship between TstcSig score and immunotherapy response in the PRJEB23709 cohort. (**G-H**) Kaplan-Meier curve showing the effect of TstcSig on progression-free survival (**G**) and overall survival (**H**) in PRJEB23709 cohort

To further demonstrate the predictive value of TstcSig in immunotherapy response, we analyzed another two immunotherapy cohorts, including a bladder cancer cohort and a melanoma cohort. In both cohorts, a higher TstcSig was associated with clinical response to immunotherapy and favorable outcomes (Fig. 6A-H). These results reinforced the association between TstcSig and immunotherapy response.

### Comparison with the 109 T cell signatures

**Fig. 7:**
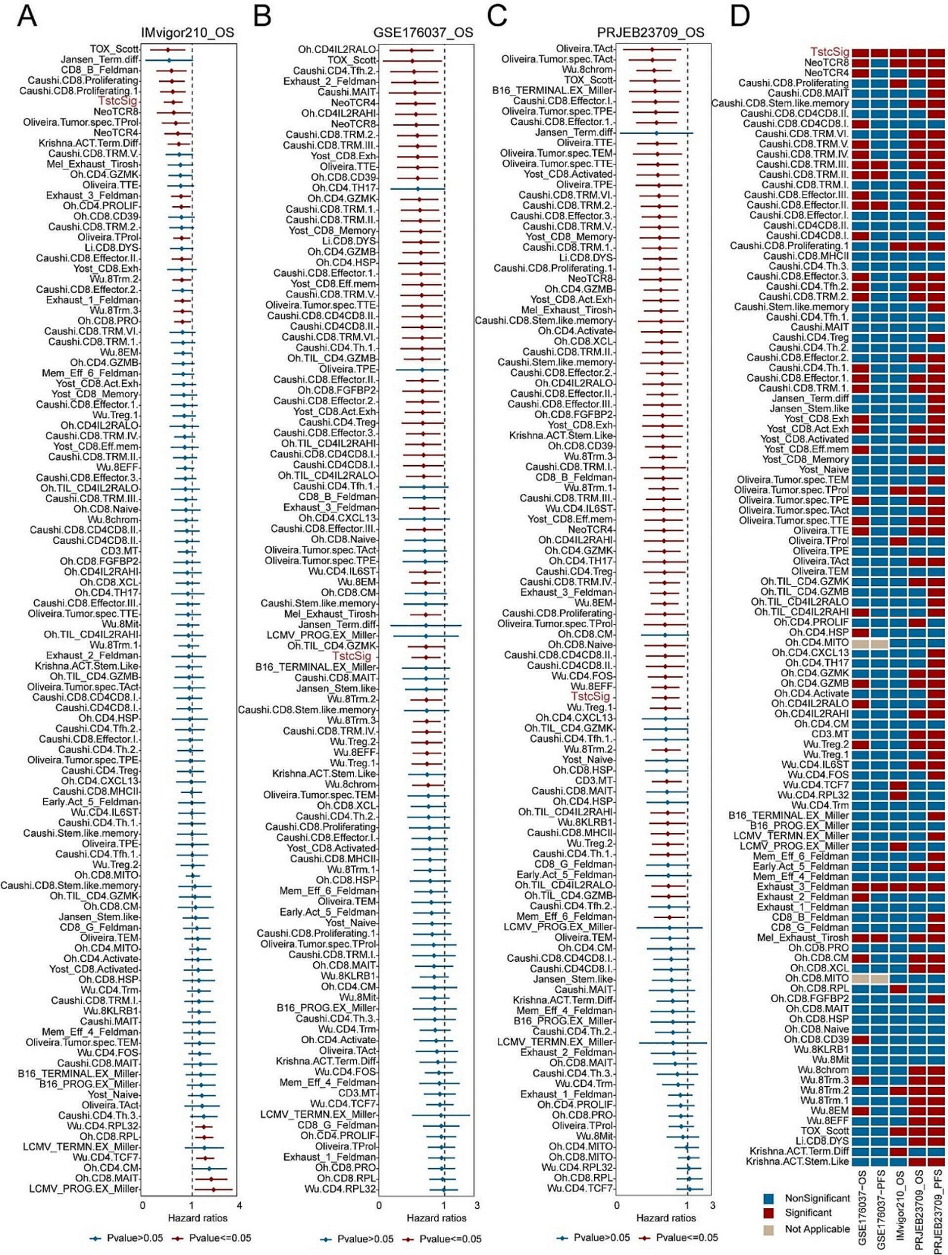
Collected 109 published transcriptome signatures of different T cell cluster for comparison with TstcSig. (**A-C**) Univariate Cox regression analysis for overall survival of TstcSig and 109 published signatures in IMvigor210 (**A**), GSE176307 (**B**), and PRJEB23709 (**C**). (**D**) Waffle diagram showing the Kaplan-Meier analysis of TstcSig and 109 published signatures. Red: Significant; Blue: Nonsignificant; Brown: Not applicable

Despite evaluating the predictive value of TstcSig, it was necessary to compare it with other T cell signatures in predicting the prognosis of patients undergoing immunotherapy. We collected 109 T cell signatures (supplementary file 1) and performed univariate Cox regression analysis for overall survival in three immunotherapy cohorts. Notably, the only TstcSig and Exhaust_3_Feldman signature were independent prognostic factors in all immunotherapy cohorts (Fig. 7A-C). Furthermore, Kaplan-Meier analysis showed that only TstcSig and Exhaust_3_Feldman signature signature maintained consistent significance in all cohorts (Fig. 7D). Overall, TstcSig displayed excellent predictive value across all cohorts compared to the other 109 T cell signatures.

### Confirmation of the correlation between TstcSig and T cells infiltration using single-cell and spatial transcriptomics

**Fig. 8:**
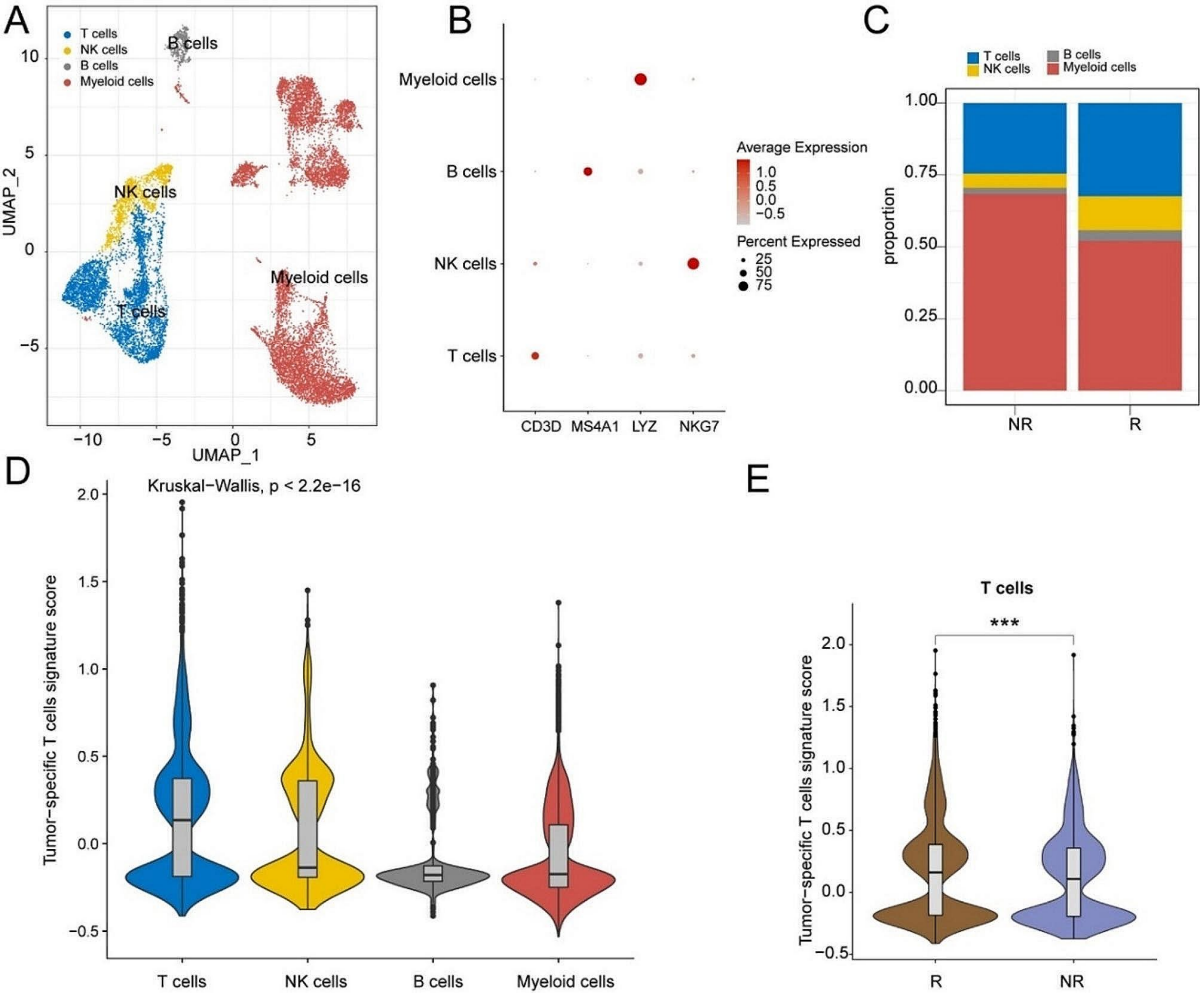
scRNA analysis of tumor infiltrating immune cells from bladder cancer patients with immunotherapy. (**A**) UMAP plot colored by different cell types. Blue: T cells; Yellow: NK cells; Grey: B cells; Red: myeloid cells. (**B**) Dot plot showing the marker gene expression of different cell types. (**C**) Bar plot showing the proportion of different cell clusters between responder and non-responder patients. (**D**) Violin plot showing TstcSig score among T cells, NK cells, B cells, and myeloid cells clusters. (**E**) Violin plot showing the relationship between TstcSig score and immunotherapy response in the GSE145281 cohort. R: respond; NR: non-respond. *** *p* < 0.001

Subsequently, we validated the correlation between TstcSig and T cell infiltration in bladder cancer using single-cell and spatial transcriptomics. Single-cell RNA sequencing data (GSE145281) of PBMCs from 10 bladder cancer patients treated with immunotherapy (5 responders and 5 non-responders) were obtained from the GEO database. After filtration, 14,468 cells, including 7,899 from responders and 6,569 from non-responders, were analyzed, identifying 4 cell types: T cells, NK cells, B cells, and myeloid cells based on the expression of markers genes (Fig. 8A-C). Notably, the T cell cluster had the highest TstcSig score among the four immune cell types (Fig. 8D). Comparing TstcSig scores between responders and non-responders revealed a significantly higher TstcSig score in T cells from responding patients (Fig. 8E).

**Fig. 9:**
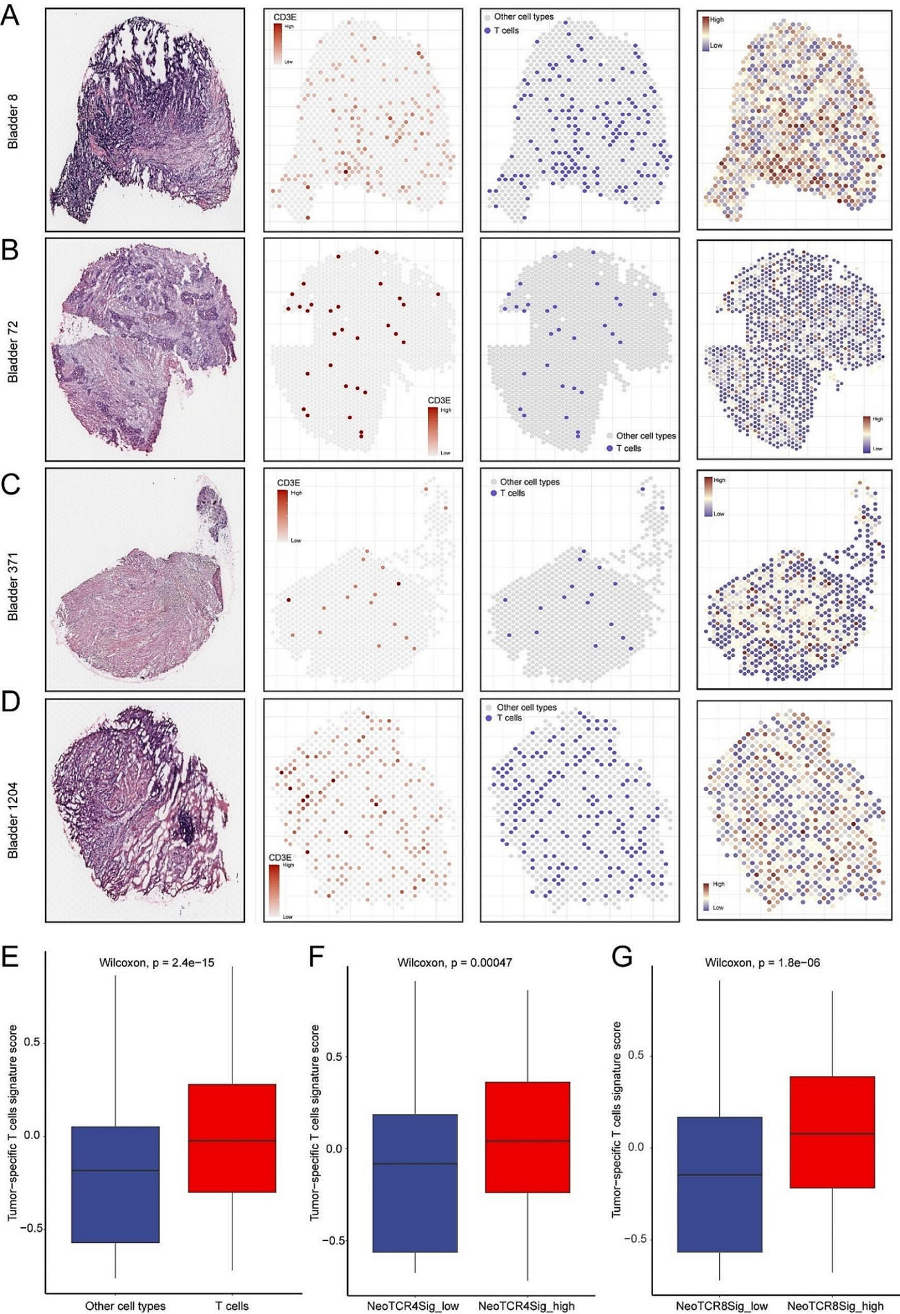
Spatial transcriptomics analysis revealing TstcSig score in T cells of bladder cancer. (**A-D**) The TstcSig score and T cell infiltration in *bladder cancer patient-8* (**A**), *bladder cancer patient-72* (**B**), *bladder cancer patient-371* (**C**) and *bladder cancer patient-1204* (**D**). (**E**) Box plot showing the relationship between TstcSig score and T cell infiltration. (**F-G**) Box plot showing the relationship between TstcSig score and NeoTCR4 (F) and NeoTCR8 signature scores (**G**)

Additionally, we included a spatial transcriptomics dataset (GSE171351) with four samples from four bladder cancer patients. TstcSig was positively related to T cell infiltration in the TME (Fig. 9A-D). For instance, *bladder cancer patient-8* with abundant T cell infiltration had a higher TstcSig score, while *bladder cancer patient-371* with less T cell infiltration had a lower TstcSig score. As expected, T cells had a significantly higher TstcSig score than other cell types in bladder cancer (Fig. 9E). Our data provided further evidence that TstcSig was significantly associated with anti-tumor neoantigen-relative T cell signatures (NeoTCR4 and NeoTCR8), indicating that TstcSig may related to neoantigen-specific T cells infiltration.

### Pan-cancer analysis of TstcSig for prognosis

**Fig. 10:**
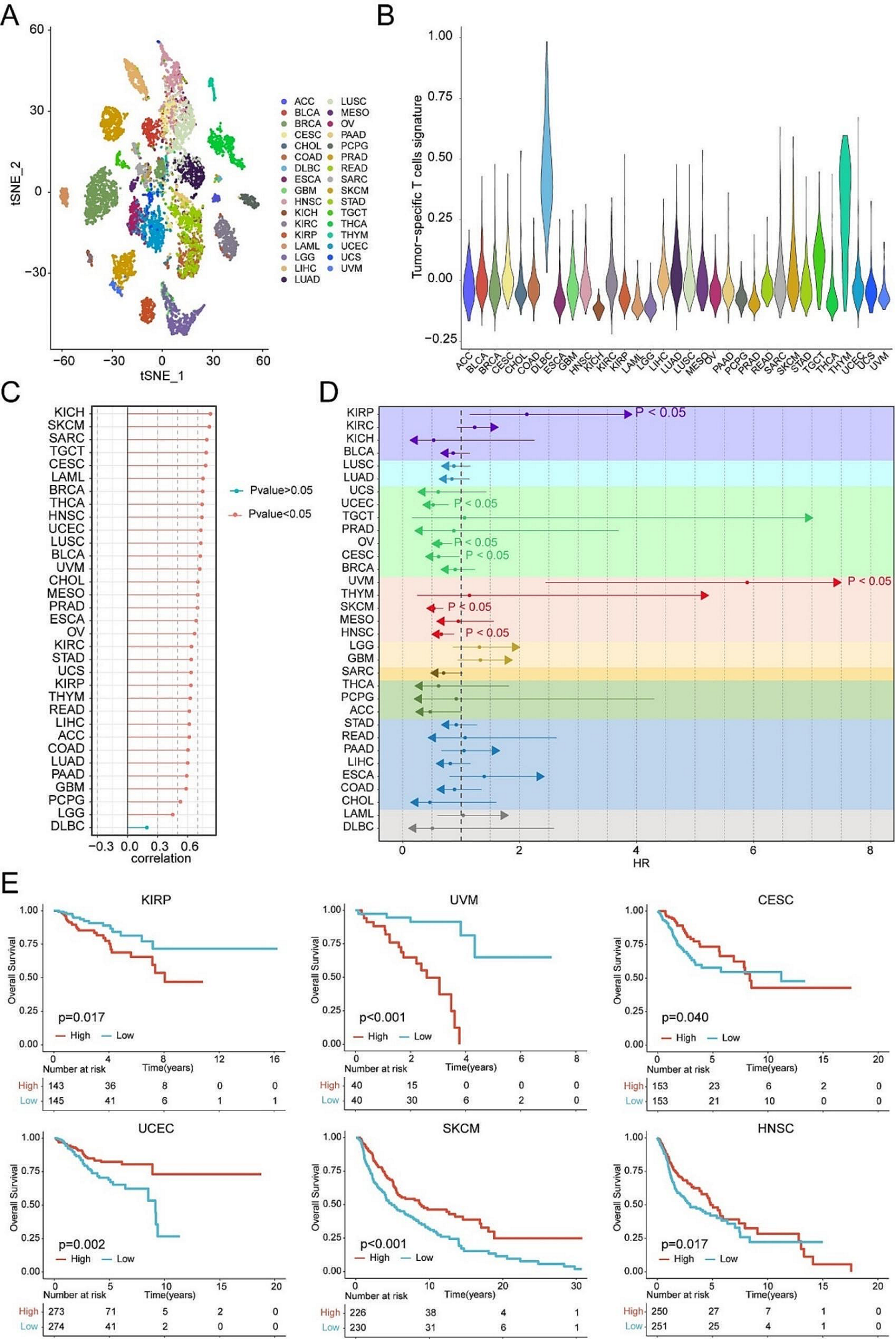
TstcSig heterogeneity and its prognostics value among human cancers. (**A**) t-SNE plots colored by 33 cancer types. (**B**) Violin plot showing TstcSig score among 33 cancer types. (**C**) Correlation analysis on TstcSig and T cell infiltration across 33 cancer types in the TCGA cohorts. (**D**) Univariate Cox regression analysis for overall survival of TstcSig among 33 cancer types. (**E**) Kaplan-Meier curve showing the effect of TstcSig on overall survival in KIRP, UVM, CESC, UCEC, SKCM, and HNSC

To explore the heterogeneity of TstcSig across different cancer types, we analyzed RNA-seq data from 10,327 tumors spanning 33 tumor types in TCGA database (Fig. 10A). The results showed significant heterogeneity of TstcSig levels, with cancers such as DLBC exhibiting higher TstcSig levels, while KICH showed lower levels (Fig. 10B). Further analysis indicated that TstcSig was positively associated with T cell infiltration in 33 cancer types, except for DLBC (Fig. 10C). Univariate Cox regression analysis was performed to evaluate the prognostic impact of TstcSig on overall survival across these cancer types. Interestingly, TstcSig was identified as a protective factor in UCEC, OV, CESC, SKCM and HNSC, while it was a risk factor in KIRP and UVM (Fig. 10D). Specifically, TstcSig was associated with adverse prognosis in KIRP and UVM, whereas it was linked to favorable prognosis in UCEC, CESC, SKCM, and HNSC (Fig. 10E). Our results highlight a positive correlation between TstcSig and T cell infiltration in most cancer types and demonstrate the heterogeneous prognostic value of TstcSig across different cancer types.

### Validation of hub gene expression and anti-tumor characteristics in tumor-specific T cells

**Fig. 11:**
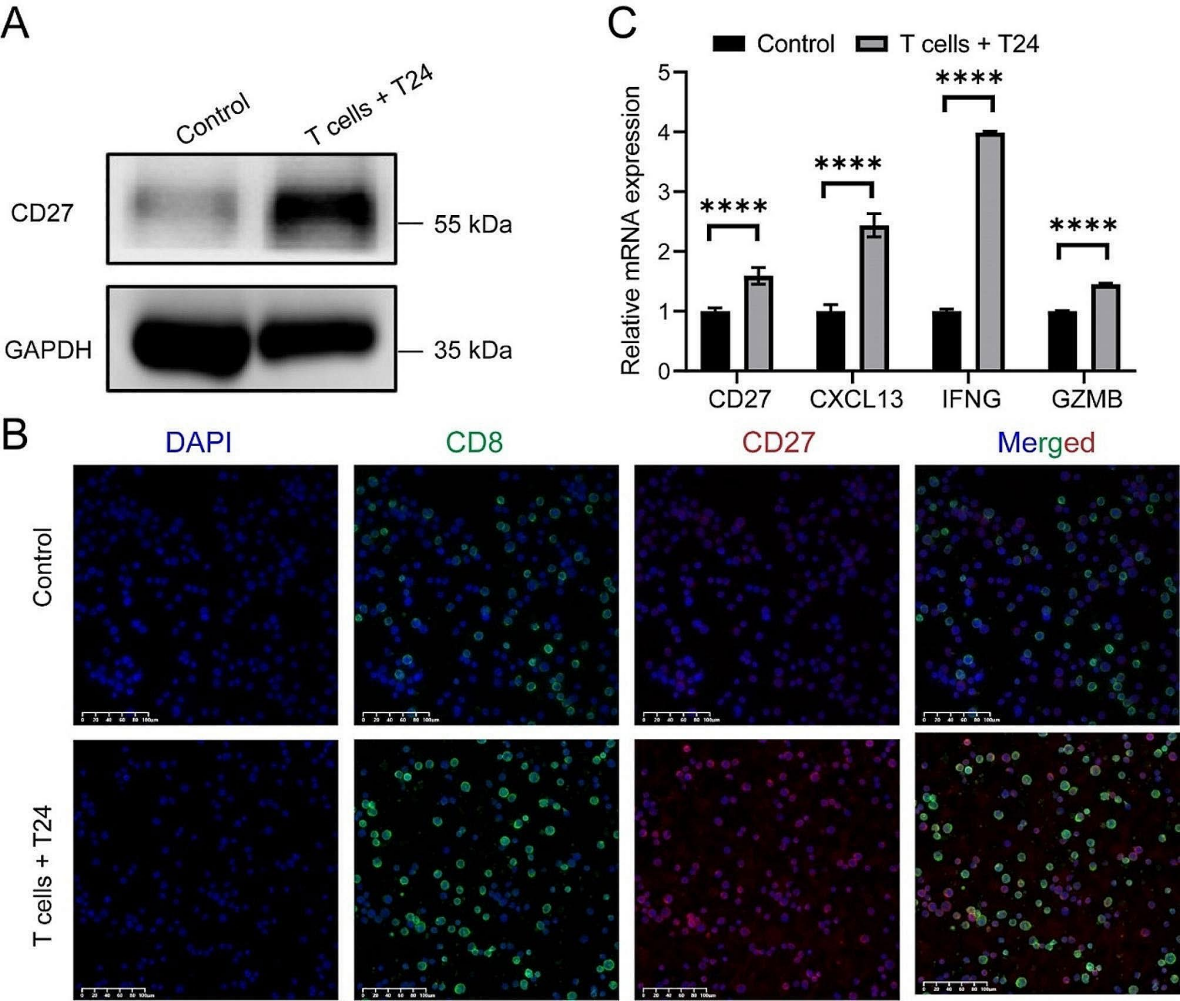
Experimental validation of the expression of CD27 in tumor-specific T cells. (**A**) The expression of CD27 in control and tumor-specific T cells was detected using Western blotting. (**B**) Representative multicolor immunofluorescence image showing the expression of CD8 and CD27 in control and tumor-specific T cell samples. (**C**) The expression of CD27, CXCL13, IFNG, and GZMB in control and tumor-specific T cell samples was detected using qRT-PCR. **** *p* < 0.0001

**Fig. 12:**
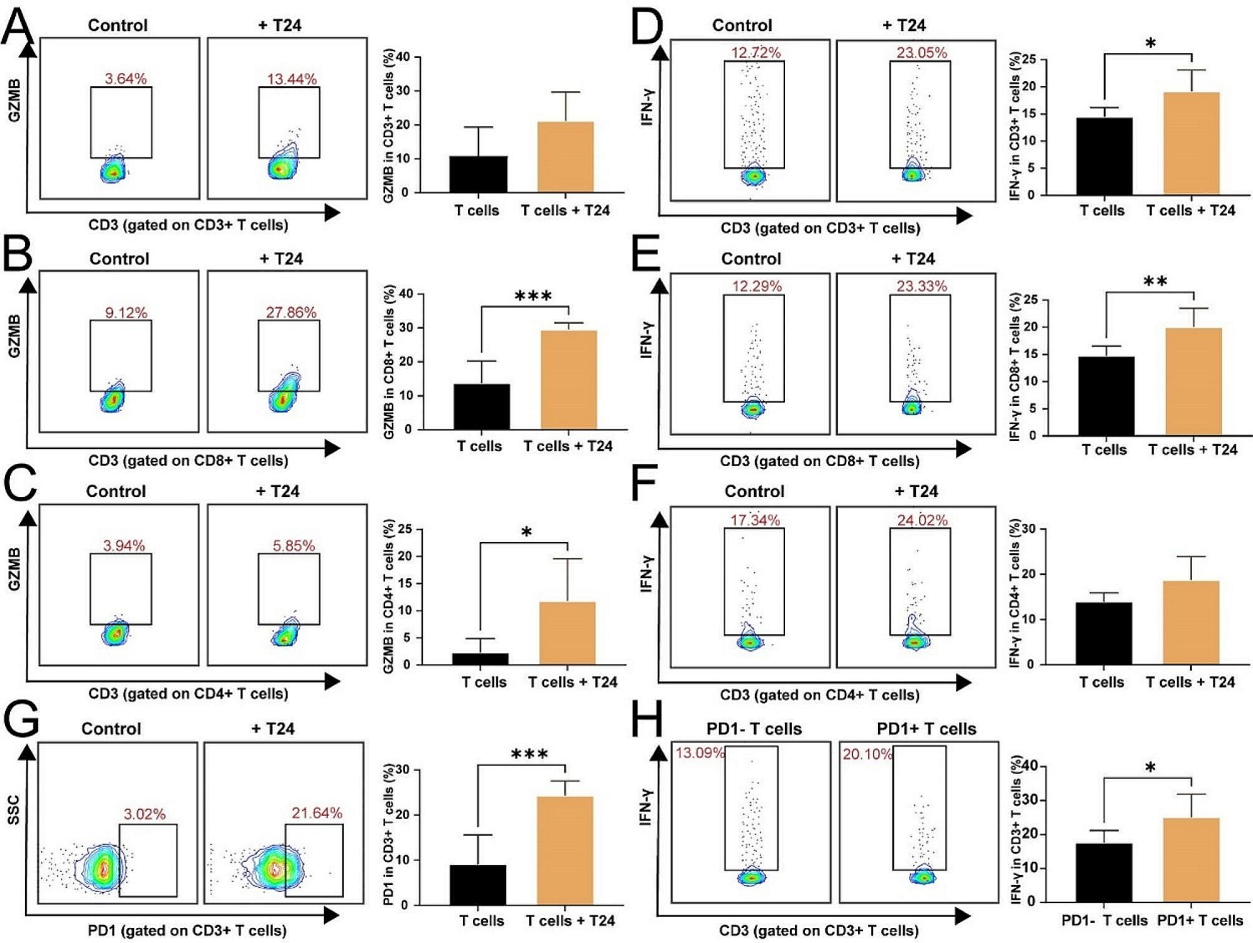
Flow cytometry analysis of characteristics in tumor-specific T cells. (**A-C**) Flow cytometry analysis of GZMB expression in vitro in CD3 + T cells (**A**), CD8 + T cells (**B**), and CD4 + T cells (**C**) co-cultured with T24 cells. (**D-F**) Flow cytometry analysis of IFN-γ expression in vitro in CD3 + T cells (**D**), CD8 + T cells (**E**), and CD4 + T cells (**F**) co-cultured with T24 cells. (**G**) Flow cytometry analysis of PD1 expression in vitro in CD3 + T cells co-cultured with T24 cells. (**H**) Flow cytometry analysis of IFN-γ expression in PD1- T cells and PD1 + T cells. * *p* < 0.05; ** *p* < 0.01; *** *p* < 0.001

To validate the results of our bioinformatics analysis, tumor-specific T cells were generated from the co-culture of tumor cell lines with PBMC assays. Western blot and immunofluorescence assays showed that the hub gene CD27 was upregulated in these tumor-specific T cells (Fig. 11A-B). Moreover, qRT-PCR results revealed significant upregulation of CD27, CXCL13, IFNG and GZMB in tumor-specific T cells using (Fig. 11C). We further measured the expression of IFN-γ, GZMB, and PD1 in tumor-specific T cells using flowcytometry. The results indicated that IFN-γ, GZMB, and PD1 were significantly upregulated in tumor-specific T cells (Fig. 12A-G). Notably, PD1 + tumor-specific T cells secreted more IFN-γ compared to PD1- tumor-specific T cells (Fig. 12H). These findings suggest that tumor-specific T cells highly express CD27 and secrete increased levels of IFN-γ and GZMB, enhancing their ability to kill tumor cells.

## Discussion

Bladder cancer is a malignant cancer with poor prognosis, contributing significantly to global morbidity and mortality [34]. In recent years, immunotherapy has become widespread in treating bladder cancer. Despite favorable responses to immunotherapy in bladder cancer, only a minority of patients benefit [6, 35, 36]. It has been reported that tumor-specific T cells are associated with cancer immunotherapy [11, 37], yet reliable biomarkers based on tumor-specific T cell-related genes for predicting immunotherapy outcomes in bladder cancer remain elusive. Here, we identified tumor-specific T cell-related genes through analysis of single-cell RNA and TCR sequencing data in bladder cancer. Subsequently, we developed a novel prognostic signature based on five tumor-specific T cell-related genes for patients undergoing immunotherapy for bladder cancer. Our data demonstrated that the TstcSig served as independent protective factor for bladder patients treated with immunotherapy. Futhermore, TstcSig strongly correlated with immune characteristics, including expression of immune checkpoint genes, tumor mutation burden and T cell infiltration. Additionally, the prognostic value of TstcSig was further validated in another two independent immunotherapy cohorts from the GEO database, exhibiting superior predictive performance compared to 109 other published T cell signatures. Pan-cancer analysis revealed heterogeneity in the prognostic value of TstcSig. In conclusion, our findings indicates that TstcSig may serve as a valuable tool for precisely selecting bladder cancer patients who are potential candidates for benefiting from immunotherapy.

The study identified several T cell subsets in the TME, such as tumor-specific T cells, virus-specific T cells, and bystander T cells [38, 39]. Extensive literature supports the close relationship between tumor-specific T cells and the efficacy of immunotherapy [40, 41]. Therefore, our study aimed to investigate the role of tumor-specific T cells in bladder cancer immunotherapy by constructing TstcSig, consisting of five tumor-specific T cell-related genes: CD27, CACYBP, TIGIT, LCK, and VAMP5. Previous studies have highlighted that the co-stimulatory molecule CD27 is associated with T cell activation and is up-regulated in tumor-specific CD4 + T cells [42]. CACYBP is linked to the G checkpoint of the cell cycle and is associated with cancer immunotherapy [43]. TIGIT has been defined as a novel target for cancer immunotherapy and is up-regulated in tumor-specific CD8 + T cells [44, 45]. LCK, as a major TCR kinase, is associated with improved overall survival in bladder cancer patients treated with immunotherapy [46]. VAMP5, a vesicle-associated membrane, plays a confirmed role immune regulation [47]. These hub genes represent potential targets for elucidating the molecular mechanisms underlying bladder cancer immunotherapy.

The association between TstcSig and immunotherapy response and prognosis was validated in two independent immunotherapy cohorts. Furthermore, the predictive value of TstcSig outperformed that of 109 other Tcell signatures. To explore potential underlying mechanisms, we stratified 298 bladder cancer patients treated with immunotherapy into high TstcSig group and low TstcSig group according to the median TstcSig score. Our data indicated that bladder cancer patients with high TstcSig exhibited “hot tumor” characteristics, characterized by a higher proportion of infiltrating immune cells, elevated immune scores, and increased expression of immune checkpoint genes such as PDCD1, CDLA4, LAG3, and CD274. Consistently, a “hot” TME signature was associated with increased response to immunotherapy [48]. Additionally, TstcSig showed significant associations with immunotherapy-related genes and immune response-related pathways, as indicated by correlation and GSEA analysis. Our findings underscored the predictive capacity of TstcSig in bladder cancer immunotherapy, linking its mechanism to the “hot” tumor immune characteristics of the TME.

To our knowledge, few studies have investigated the prediction of immunotherapy outcomes based on tumor-specific T cell-related genes in bladder cancer. Our study utilized multi-omics data and large datasets to discover and validate TstcSig in predicting immunotherapy response. However, this study is limited by its reliance on public datasets and the absence of additional experimental validation. Moreover, the analysis was confined to the transcriptome level, warranting further research and validation across other omics domains. The role of CD27 in T cells and its underlying molecular mechanisms require comprehensive elucidation.

In conclusion, we have developed and validated a novel tumor-specific T cell signature (including VAMP5, TIGIT, LCK, CD27, and CACYBP) as a prognostic biomarker for bladder cancer patients treated with immunotherapy using single cell multi-omics data.

### Electronic supplementary material

Below is the link to the electronic supplementary material.

Supplementary Material 1

## Data Availability

No datasets were generated or analysed during the current study.

## Abbreviations

GEO: Gene expression omnibus
TstcSig: Tumor-specific T cell signature
TCGA: The cancer genome atlas
TCR: T cell receptor
PCA: Principal component analysis
UMAP: Uniform Manifold Approximation and Projection
DEGs: differentially expressed genes
GO: Gene Ontology
KEGG: Kyoto Encyclopedia of Genes and Genomes
TME: Tumor microenvironment
GSVA: Gene set variation analysis
OS: Overall survival
PFS: Progression-free survival
TMB: Tumor-mutation burden
TNB: tumor neoantigen burden
PBMC: Peripheral blood mononuclear cells
ACC: Adrenocortical Carcinoma
BLCA: Bladder Urothelial Carcinoma
BRCA: Breast invasive carcinoma
CESC: Cervical squamous cell carcinoma and endocervical adenocarcinoma
CHOL: Cholangio carcinoma
COAD: Colon adenocarcinoma
DLBC: Lymphoid Neoplasm Diffuse Large B-cell Lymphoma
ESCA: Esophageal carcinoma
GBM: Glioblastoma multiforme
HNSC: Head and Neck squamous cell carcinoma
KICH: Kidney Chromophobe
KIRC: Kidney renal clear cell carcinoma
KIRP: Kidney renal papillary cell carcinoma
LAML: Acute Myeloid Leukemia
LGG: Brain Lower Grade Glioma
LIHC: Liver hepatocellular carcinoma
LUAD: Lung adenocarcinoma
LUSC: Lung squamous cell carcinoma
MESO: Mesothelioma
OV: Ovarian serous cystadenocarcinoma
PAAD: Pancreatic adenocarcinoma
PCPG: Pheochromocytoma and Paraganglioma
PRAD: Prostate adenocarcinoma
READ: Rectum adenocarcinoma
SARC: Sarcoma
SKCM: Skin Cutaneous Melanoma
STAD: Stomach adenocarcinoma
TGCT: Testicular Germ Cell Tumors
THCA: Thyroid carcinoma
THYM: Thymoma
UCEC: Uterine Corpus Endometrial Carcinoma
UCS: Uterine Carcinosarcoma
UVM: Uveal Melanoma
